# Supplementary figures and images for: HIV-1 Vpu Promotes Phagocytosis of Infected CD4+ T Cells by Macrophages through Downregulation of CD47
Source: mBio. 2021 Aug 24;12(4):e01920-21. doi: 10.1128/mBio.01920-21 (PMC8406190; doi:10.1128/mBio.01920-21)

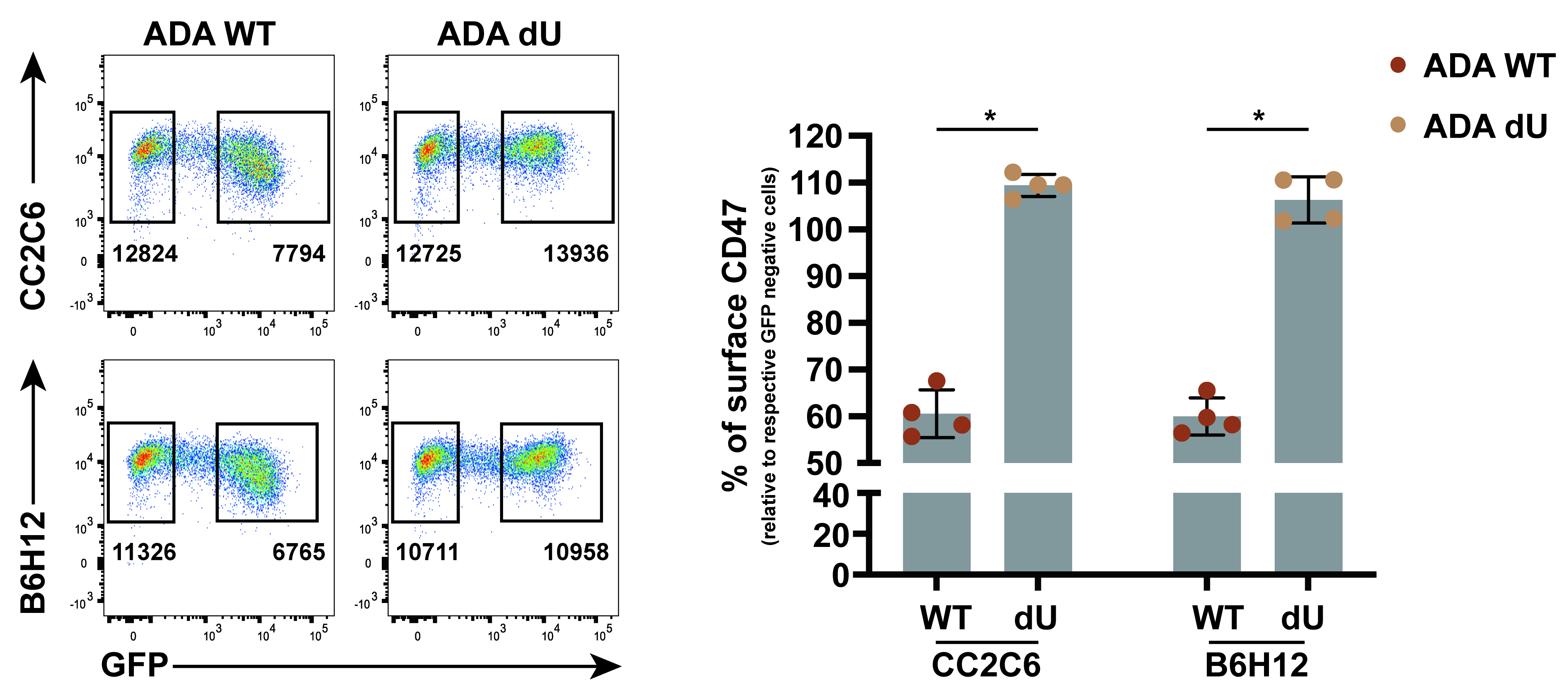

Supplement: FIG S1 [file mbio.01920-21-sf001.tif]

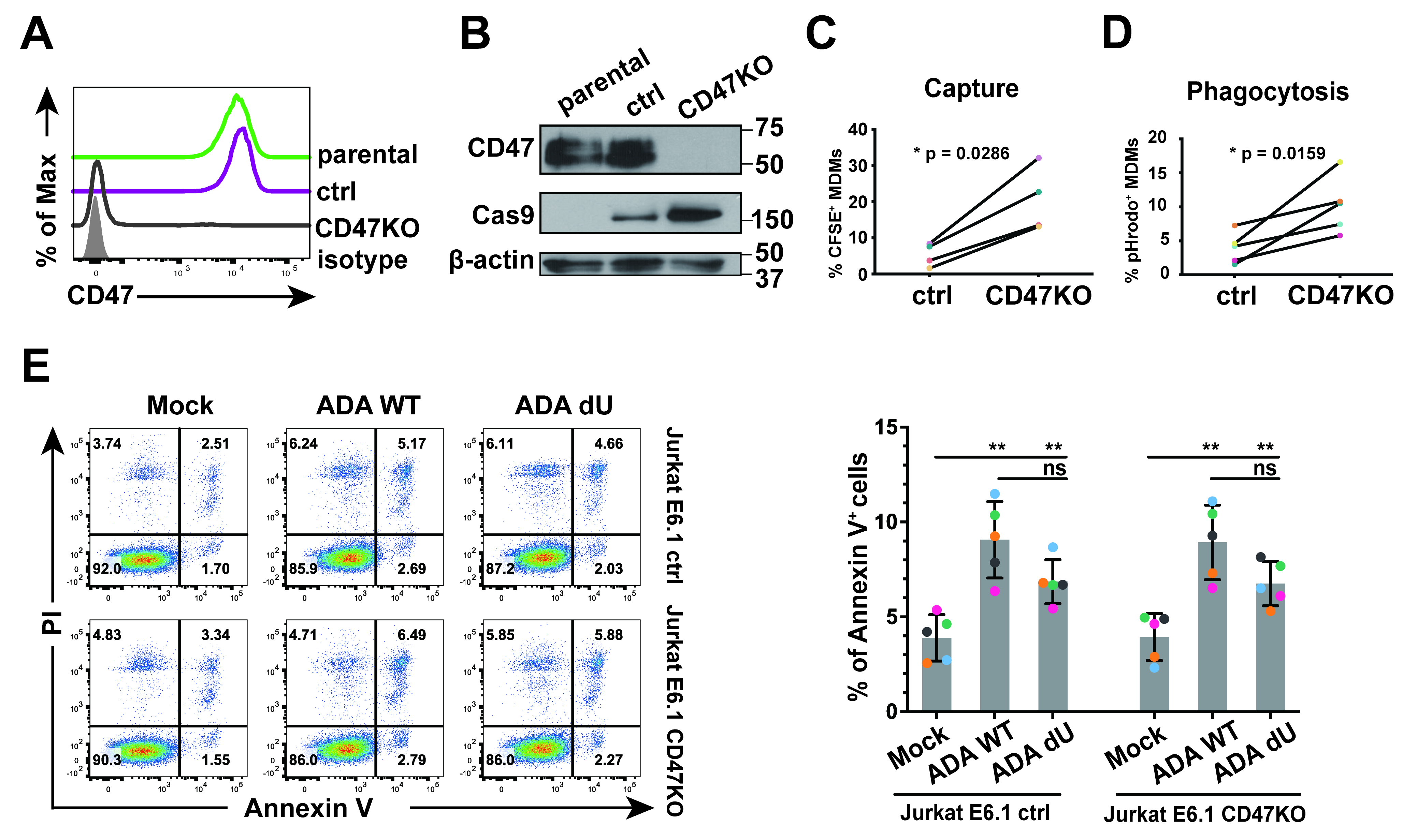

Supplement: FIG S2 [file mbio.01920-21-sf002.tif]

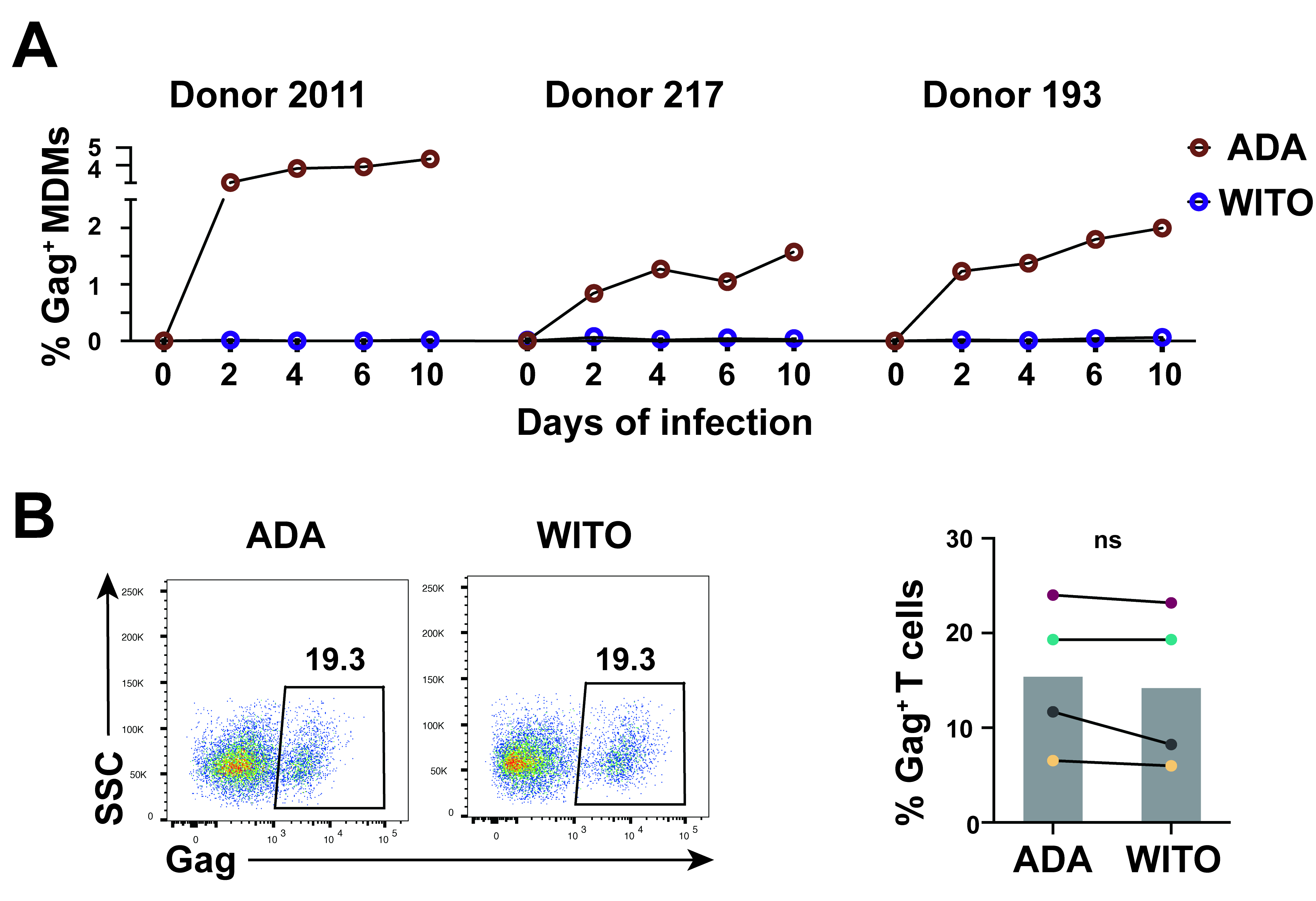

Supplement: FIG S3 [file mbio.01920-21-sf003.tif]

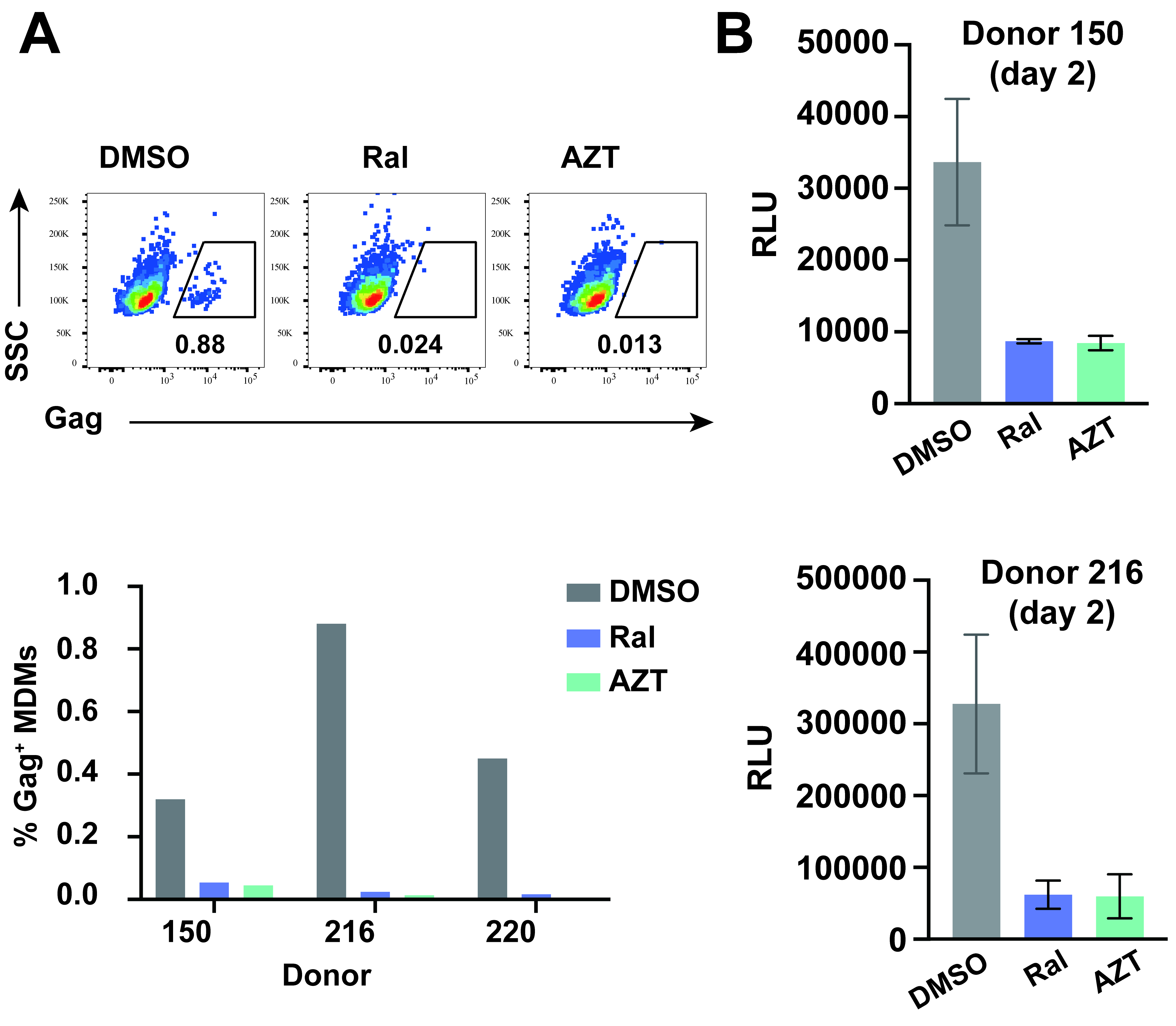

Supplement: FIG S4 [file mbio.01920-21-sf004.tif]

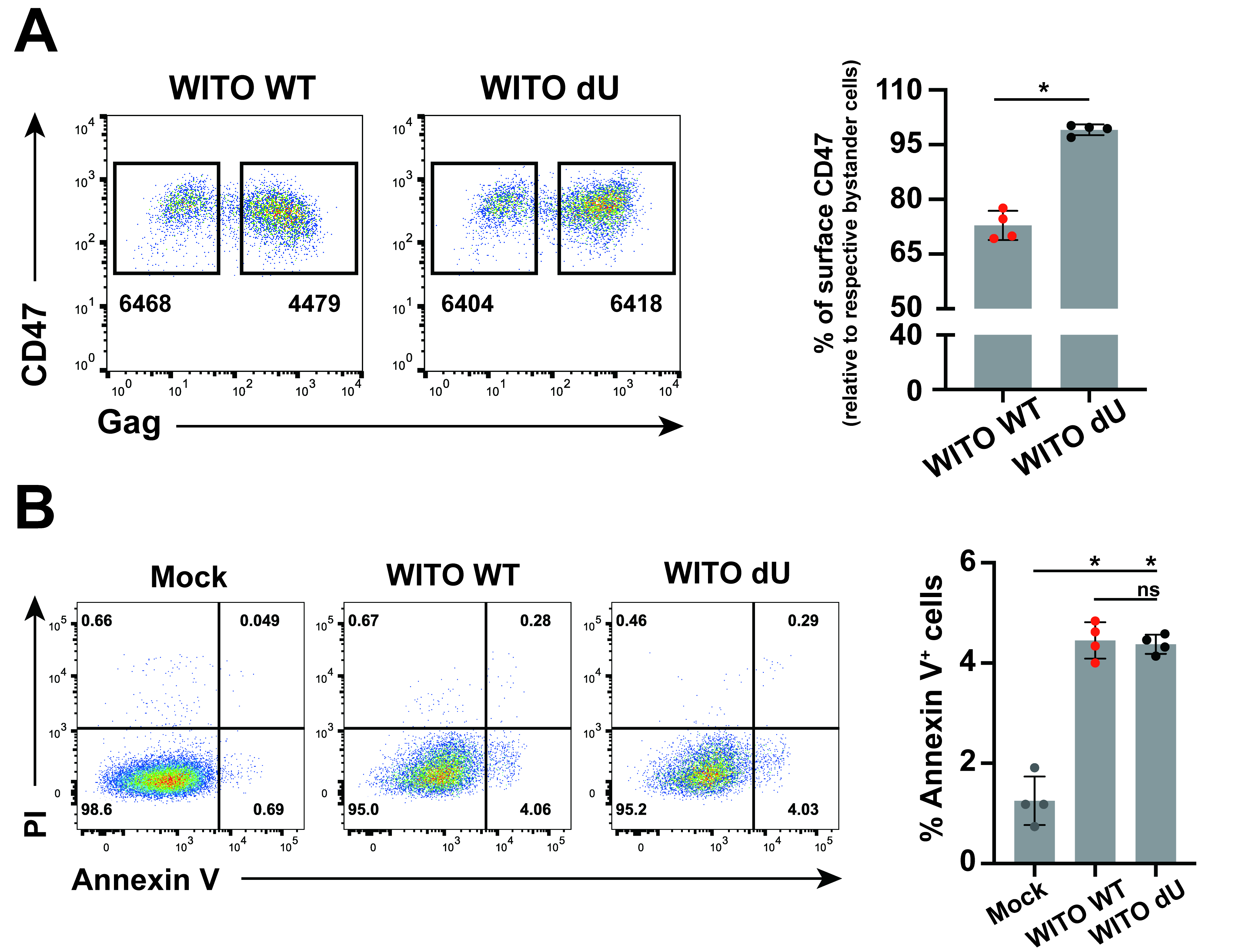

Supplement: FIG S5 [file mbio.01920-21-sf005.tif]

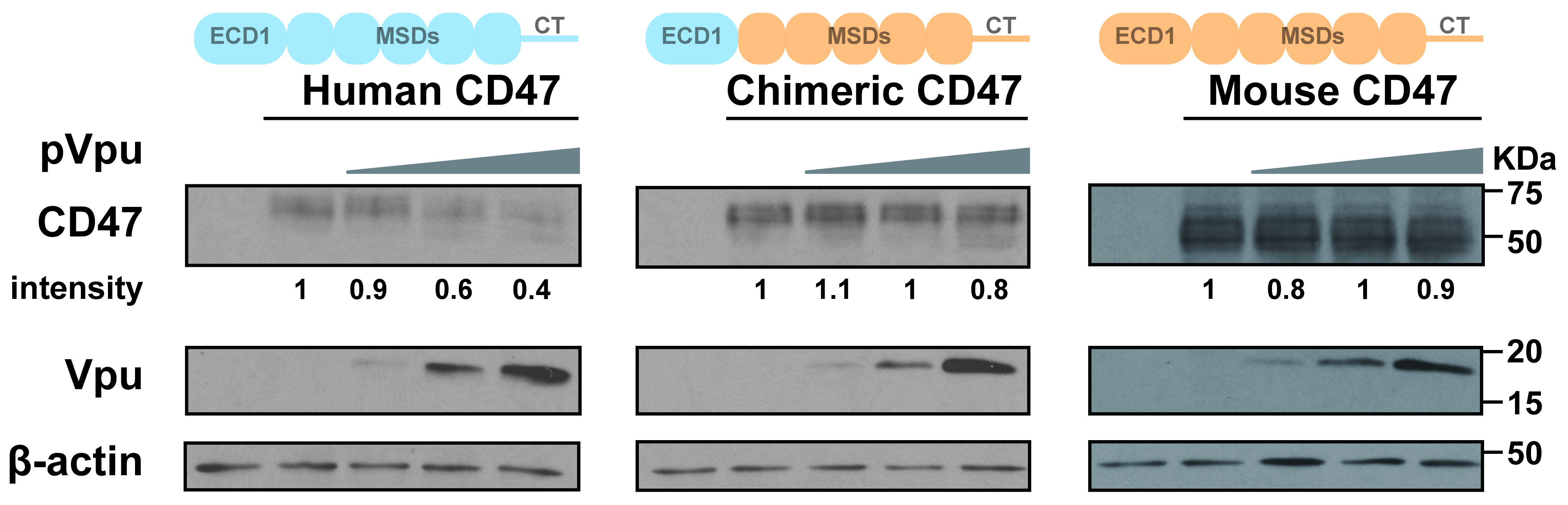

Supplement: FIG S6 [file mbio.01920-21-sf006.tif]

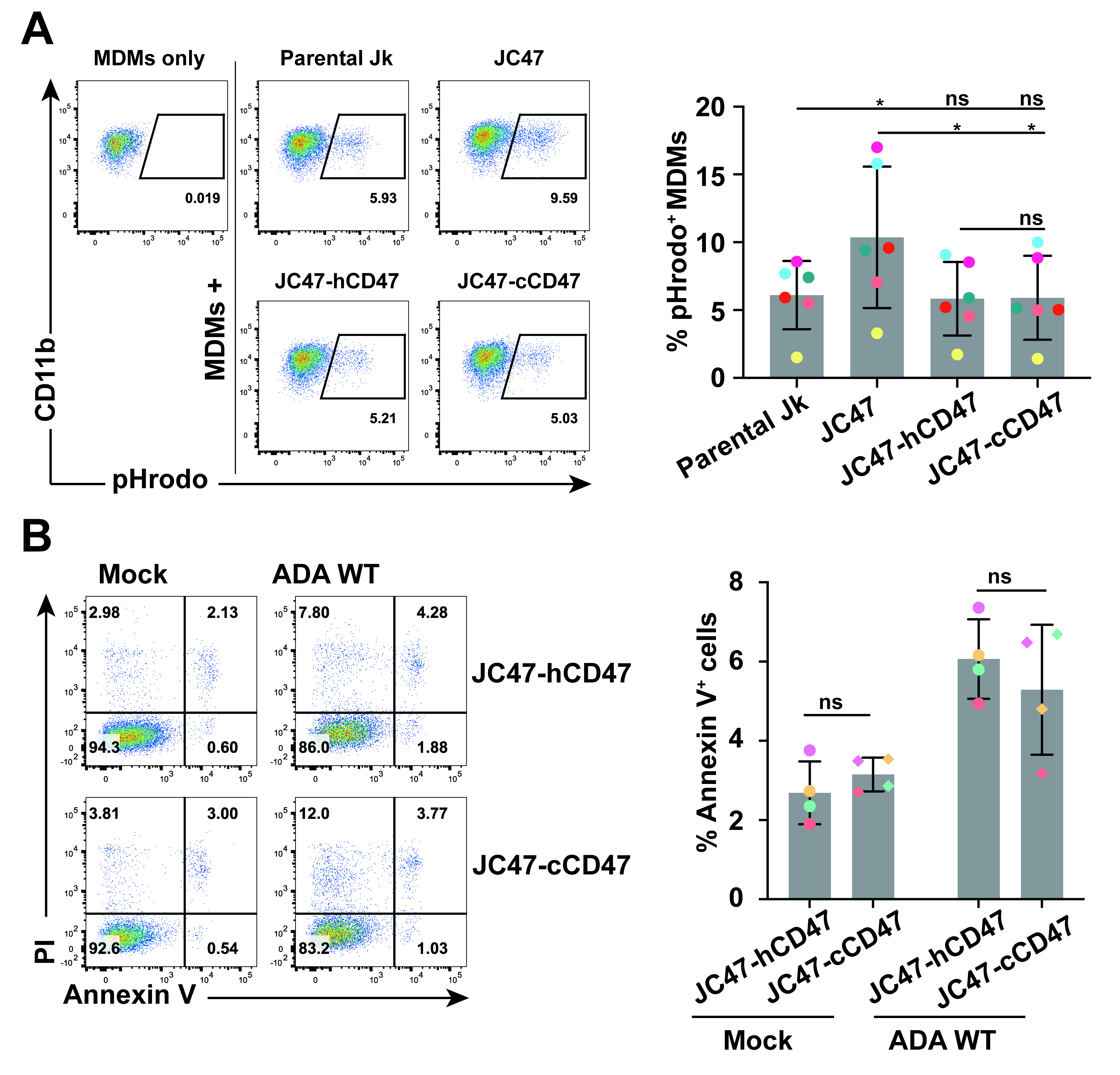

Supplement: FIG S7 [file mbio.01920-21-sf007.tif]
