# Supplementary material for: HIV-1 Vpu Promotes Phagocytosis of Infected CD4+ T Cells by Macrophages through Downregulation of CD47
Source: mBio. 2021 Aug 24;12(4):e01920-21. doi: 10.1128/mBio.01920-21 (PMC8406190; doi:10.1128/mBio.01920-21)
Supplement: TABLE S1 [file mbio.01920-21-st001.pdf]

Table S1. Oligonucleotides used in this study

Designation	Oligonucleotide sequence (5'-to-3')	Purpose
ADA Vpu A15L forward	GTAGCATTAGTAGTATTAGCAATAATAGCAATAGTTG	Vpu TMD (A_{15}) mutation
ADA Vpu A15L reverse	CAACTATTGCTATTATTGCTAATACTACTAATGCTAC	Vpu TMD (A_{15}) mutation
ADA Vpu W23A forward	GCAATAATAGCAATAGTTGTGGCGACCATAGTATTCATAG	Vpu TMD (W_{23}) mutation
ADA Vpu W23A reverse	CTATGAATACTATGGTCGCCACAACTATTGCTATTATTGC	Vpu TMD (W_{23}) mutation
ADA Vpu S53/57A forward	TAACAGAAAGAGCAGAAGACGCTGGCAATGAAGCTGAAGGGGATCAGGAAGAAT	Vpu $DS_{53}GNES_{57}$ mutations
ADA Vpu S53/57A reverse	ATTCTTCCTGATCCCCTTCAGCTTCATTGCCAGCGTCTTCTGCTCTTTCTGTTA	Vpu $DS_{53}GNES_{57}$ mutations
ADA Vpu AxxxAV forward	TGAAGGGGATCAGGAAGCATTATCAGCAGCTGTGGAAATGGGGCATC	Vpu $E_{63}xxxL_{67}V$ mutations
ADA Vpu AxxxAV reverse	GATGCCCCATTTCCACAGCTGCTGCTGATAATGCTTCCTGATCCCCTTCA	Vpu $E_{63}xxxL_{67}V$ mutations
EcoRI forward for WITO dU	TAATAAGAATTCTGCAACAACTGCTGTTTATT	Vpu-defective mutation
delU reverse for WITO dU	TGGTCCACACAACTAAAAGAAAGCATTACATATATT	Vpu-defective mutation
delU forward for WITO dU	AATATATGTAATGCTTTCTTTTAGTTGTGTGGACCA	Vpu-defective mutation
BamHI reverse for WITO dU	ACTTCTGGATCCCCTCCTGAGGATTGGTTAAA	Vpu-defective mutation
HindIII forward for human CD47-HA	CCAAGCTTATGTGGCCCCTGGTAGCGGCG	HA-tag for human CD47
BamHI reverse for human CD47-HA	CGGGATCCTTAAGCGTAATCTGGAACATCGTATGGGTAGTTATTCCTAGGAGGT	HA-tag for human CD47
Human CD47 forward	AGCTCGTCGACAAGCTTATGT	Chimeric CD47
Mouse CD47 reverse	GTTCTAGAGGATCCTTCAAGCGTAA	Chimeric CD47
Forward for human - mouse chimeric CD47	TGGTTTTCTCCAAATGAAAA	Chimeric CD47
Reverse for human- mouse chimeric CD47	TTTTCATTTGGAGAAAACCA	Chimeric CD47
XmaI forward for hCD47 into pWPI	ACATCCCGGGATGTGGCCCCTGGTA	pWPI-hCD47 lentivector
BamHI reverse for hCD47 into pWPI	AAGGGATCCTTAGTTATTCCTAGGAGG	pWPI-hCD47 lentivector
BamHI reverse for cCD47 into pWPI	AAGGGATCCTTACCTATTCCTAGGAGG	pWPI-cCD47 lentivector
